# Coronary artery bypass grafting vs. drug-eluting stent implantation in patients with end-stage renal disease requiring dialysis

**DOI:** 10.1080/0886022X.2019.1710187

**Published:** 2020-01-09

**Authors:** Zhi Wang, Yanjun Gong, Fangfang Fan, Fan Yang, Lin Qiu, Tao Hong, Yong Huo

**Affiliations:** Department of Cardiology, Peking University First Hospital, Beijing, China

**Keywords:** Coronary heart disease, dialysis-dependent CKD, PCI, CABG

## Abstract

**Objectives:**

To evaluate the optimal revascularization strategy for patients with coronary artery disease (CAD) and end stage renal disease (ESRD) in the drug-eluting stent (DES) era.

**Methods:**

One hundred and twelve patients with ESRD treated with coronary artery bypass grafting (CABG) or percutaneous coronary intervention (PCI) were enrolled from 2007 to 2017. All patients were dialysis-dependent, of which 26 received CABG and 86 underwent PCI. The primary endpoint was all-cause mortality. Secondary endpoints were major adverse cardiovascular events including myocardial infarction, stroke, repeat revascularization, and death.

**Results:**

The CABG group had a higher prevalence of left main CAD (57.7% vs. 11.6%, *p* < .01) compared with PCI group. The short-term (within 30 days after the procedure) risk of death was higher in CABG group compared with PCI group (15.4% vs. 1.2%, *p* < .05). The two groups exhibited similar rate of primary endpoints (50.0% vs. 40.7%, *p* = .37) and secondary endpoints (65.4% vs. 60.5%, *p* = .97) in long-term observation. Multivariate Cox regression showed that patients older than 65 or underwent peritoneal dialysis (PD) had significant higher rate of mortality than those under 65 (HR 2.85; 95% CI 1.20–6.85; *p* < .05) or underwent hemodialysis (HD) (HR 6.69; 95% CI 2.35–19.05; *p* < .01).

**Conclusions:**

Among patients with CAD and dialysis-dependent chronic kidney disease (CKD), treatment with CABG or PCI with DES exhibited similar long-term outcomes. However, CABG was associated with higher short-term risk of death. Higher mortality was revealed in patients over 65 years and underwent PD.

## Introduction

Cardiovascular disease is the leading cause of death in patients with chronic kidney disease (CKD), especially in patients with end-stage renal disease (ESRD) requiring dialysis [1,2]. According to the United States Renal Data System (USRDS), in dialysis patients, the annual rate of myocardial infarction and/or angina pectoris was 10% and all-cause mortality was 23.6% per year, of which cardiac disease accounting for 45% [3]. Despite the high risk of coronary artery disease (CAD), the current evidence for optimal revascularization strategy was predominantly based on observational studies instead of randomized clinical trials. In the bare-metal stent (BMS) era, some observational studies comparing coronary artery bypass grafting (CABG) and percutaneous coronary intervention (PCI) have suggested that CABG might have better long-term survival rate than PCI [4,5]. Thus, the ESC guidelines recommend CABG over PCI in patients with ESRD and triple vessel CAD, based on the data derived from these studies [6,7]. The advance of drug-eluting stents (DES) and anti-platelet or metabolic control treatment has made PCI more reliable than ever before, which has dramatically reduced restenosis rate and improved clinical outcome [8]. Meanwhile, the relative benefit of CABG is compromised by the risks of surgical complications. Therefore, we assess the short-term and long-term rates of major adverse events and survival rate in dialysis-dependent ESRD patients treated with CABG or PCI with DES in this retrospective, nonrandomized analysis.

## Materials and methods

We identified 112 dialysis-dependent CKD patients who underwent either CABG or DES-PCI for CAD at Peking University First Hospital from 1 April 2007 to 1 June 2017. Dialysis-dependent CKD was defined as estimated glomerular filtration rate <15 mL/min/1.73 m^2^ which was calculated by the simplified MDRD equation, and all patients had been on dialysis for at least 1 month before revascularization. The revascularization strategy was decided after coronary angiography and was depend on discussion of the heart team and the willing of the family. The PCI procedure was performed by skilled operators using DES. During the CABG procedure, internal mammary artery grafts were preferentially utilized, and complete revascularization was always attempted. Clinical data, coronary artery characteristics, and procedural data were collected for all patients. Follow-up was conducted in outpatient clinics and by phone-calls. This study was approved by the Ethics Committee of Peking University First Hospital with the approval number 2019 (research) 324.

The primary endpoint of the study was short-term (within 30 days after the procedure) and long-term all-cause mortality. Secondary endpoints were long-term major adverse cardiovascular events (MACEs) including myocardial infarction, stroke, repeat revascularization, or death.

Baseline variables of the patients between the two treatment groups were compared with the chi-square statistics for categorical variables and the *t* test for continuous variables. Fisher’s exact test was used for categorical variables with nominal scales and the Wilcoxon rank-sum test for those with ordinal scales. Survival curves were constructed using the Kaplan–Meier estimates and were compared with the log-rank test. Adjusted Cox proportional hazard models were used to assess the short-term and long-term rates of clinical outcomes between the two treatment strategies. In the multivariate models, adjusted covariates included age, gender, body mass index (BMI), choice of dialysis modality, number of diseased vessels, involvement of left main disease and LVEF. All reported *p* values are two-sided, and *p* values<.05 were considered statistically significant. The statistical analysis was performed with Empowerstates (X&Y Solutions, Inc. Boston, MA) and R3.4.3 (http://www.R-project.org).

## Results

### Baseline characteristics

The baseline characteristics according to treatment strategy are shown in Table 1. The mean age of patients was 62.5 years, 69.6% of the patients were men, and 54% patients had diabetes mellitus. In the PCI group, 211 diseased arteries were revealed and 205 DES were implanted, among which nine stents were first-generation DES and 196 were second or third-generation ones. Among the CABG group, left internal mammary artery grafts were used in 20 out of 26 patients (76.9%) and 25 of the operation (96.2%) were underwent off-pump. There was no significant differences observed between CABG and PCI groups in gender, age, BMI, LVEF measured by echocardiography, prevalence of current smokers and previous history of myocardial infarction, hypertension, and diabetes mellitus. There were also no significant differences in history of PCI or CABG procedures between the two groups. When it came to the method of dialysis (hemodialysis (HD) or peritoneal dialysis (PD)), there was no difference between two groups, either. The percentage of acute coronary syndrome patients and number of diseased vessels showed no difference, but the patients in CABG group had a higher incidence of LM disease (57.7% vs. 11.6%, *p* < .01) compared to PCI group. As for medical therapy, the use of platelet P2Y12 receptor inhibitor (100% vs. 26.9%, *p* < .01), Renin–angiotensin–aldosterone system (RAAS) inhibitor (41.9% vs. 15.4%, *p* < .05) and statin (91.9% vs. 46.2%, *p* < .01) was higher in the PCI group than the CABG group.

**Table 1.** Baseline characteristics based on treatment strategies^a^.

	PCI (*n* = 86)	CABG (*n* = 26)	*p* Value
Male gender (% of patients)	67.4%	76.9%	.357
Age (years)	62.17 ± 11.30	63.65 ± 10.85	.556
Hypertension (%)	96.5%	96.2%	1.0
Diabetes mellitus (%)	54.7%	50.0%	.677
Current smoker (%)	11.8%	23.1%	.264
Previous MI (%)	23.3%	19.2%	.666
Previous PCI (%)	10.5%	7.7%	.968
Previous CABG (%)	1.2%	0%	1.0
Dialysis modality (% of hemodialysis)	54.7%	61.5%	.535
ACS (%)	87.2%	100%	.123
Body mass index	24.26 ± 3.67	24.37 ± 3.34	.797
One vessel disease (%)	15.1%	0%	.079
Two vessels disease (%)	24.4%	19.2%	.583
Three vessels disease (%)	60.5%	80.8%	.057
LM disease (%)	11.6%	57.7%	<.01
LVEF (%)	54.4 ± 14.5	56.9 ± 17.7	.524
Medical therapy			
Aspirin (%)	91.9%	96.2%	.756
P2Y12 receptor inhibitor (%)	100%	26.9%	<.01
RAAS inhibitor (%)	41.9%	15.4%	.014
β blocker (%)	84.9%	84.6%	1.0
Statin (%)	91.9%	46.2%	<.01

ACS: acute coronary syndrome; CABG: coronary artery bypass graft; MI: myocardial infarction; LM: left main; LVEF: left ventricle ejection fraction; RAAS: renin–angiotensin–aldosterone system.

a Data presented as percent or as the mean value ± SD.

### Clinical outcomes after revascularization

**Figure 1.**
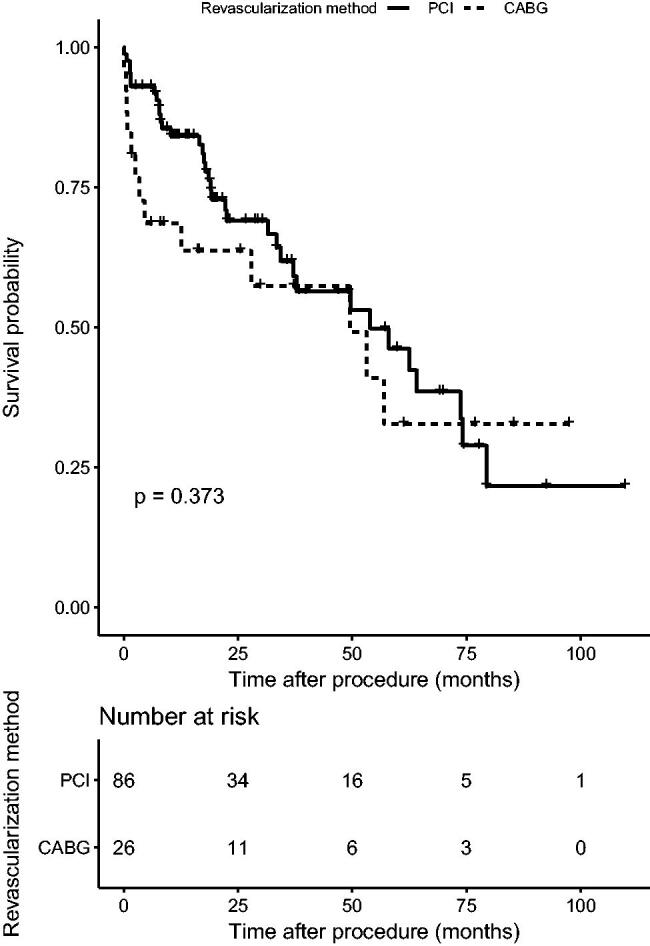
Overall survival between CABG and PCI.

**Figure 2.**
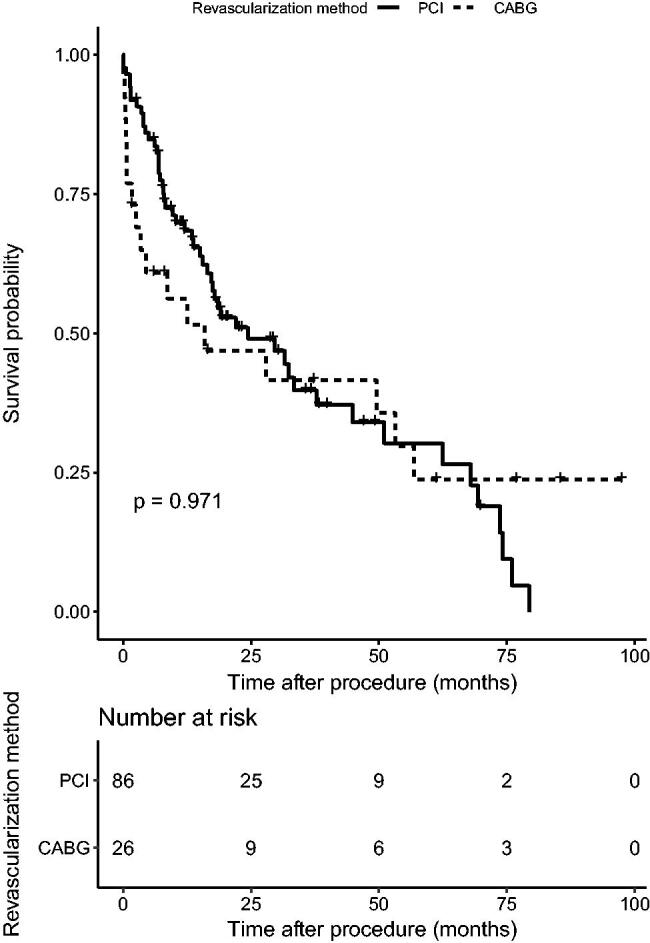
MACE-free survival between CABG and PCI.

**Figure 3.**
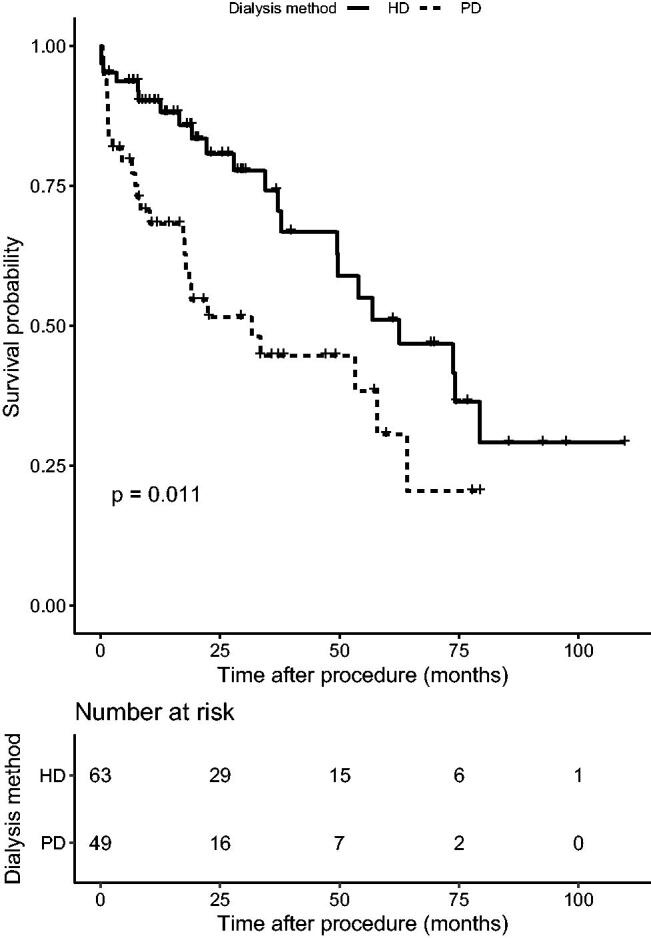
Survival between different dialysis modality.

During the 19.3 month follow-up period (interquartile range (IQR)=8.14–38.76), primary outcome (all-cause mortality) occurred in 35 (40.7%) patients in the PCI group and 13 (50.0%) patients in the CABG group (hazard ratio (HR), 1.34; 95% confidence interval (CI), 0.71–2.53; *p* = .37). Twenty-two (45.8%) patients suffered cardiac death in CABG and PCI groups (30.8% vs. 51.4%, *p*>.05). Secondary outcomes occurred in 52 (60.5%) patients in the PCI group and 17 (64.5%) patients in the CABG group (HR, 1.01; 95% CI, 0.58–1.77; *p* = .97) (Figures 1 and 2). In the PCI group, there were 62 secondary endpoint events, among which 15 were myocardial infarction, two were stroke, 21 were revascularization (among which 16 were in stent restenosis), and 24 were death. At the same time, the CABG group revealed 19 secondary endpoint events, among which four stroke, two revascularization and 13 death. A higher incidence of short-term death was observed in CABG group (15.4% vs. 1.2%, *p* < .05). The Kaplan–Meier curve did not show a significant difference in both primary and secondary outcomes between two groups. Multivariate Cox regression, which included revascularization option, age, gender, BMI, history of diabetes, current smoker, dialysis modality, number of diseased vessels, involvement of left main disease and LVEF, showed that patients older than 65 or underwent PD were associated with significant higher mortality than those under 65 (HR 2.87; 95% CI 1.20–6.85; *p* < .05) or were treated with HD (HR 6.69; 95% CI 2.35–19.05; *p* < .01) (Table 2, Figure 3).

**Table 2.** Multivariate Cox regression of mortality^a^.

Exposure	Univariate	Multivariate
Revascularization method		
PCI	1.0	1.0
CABG	1.34 (0.71, 2.53) .37	1.65 (0.51, 5.31) .40
Age		
<65	1.0	1.0
≥65	3.19 (1.77, 5.74) <.01*	2.87 (1.20, 6.85) .02*
Gender		
Male	1.0	1.0
Female	1.49 (0.83, 2.67) .18	1.27 (0.51, 3.16) .61
BMI	1.12 (1.03, 1.22) .01	1.06 (0.96, 1.18) .25
Diabetes		
No	1.0	1.0
Yes	1.01 (0.57, 1.78) .98	0.61 (0.25, 1.51) .29
Current smoker		
No	1.0	1.0
Yes	0.63 (0.25, 1.60) .33	1.07 (0.35, 3.28) .90
Dialysis modality		
Hemodialysis	1.0	1.0
Peritoneal dialysis	2.12 (1.19, 3.77) .01*	6.69 (2.35, 19.05) <.01*
Diseased vessel no.		
One	1.0	1.0
Two	0.72 (0.26, 1.99) .52	1.30 (0.31, 5.41) .72
Three	0.79 (0.33, 1.91) .61	1.24 (0.32, 4.75) .76
Presence of LM disease		
No	1.0	1.0
Yes	1.41 (0.74, 2.67) .30	2.27 (0.67, 7.63) .19
LVEF		
≥50%	1.0	1.0
<50%	1.23 (0.68, 2.24) .49	0.91 (0.38, 2.22) .84

HD: hemodialysis; LM: left main; LVEF: left ventricle ejection fraction; PD: peritoneal dialysis.

a Data: HR (95% CI) *p* value.

* *p*<.05.

## Discussion

There were increasing number of CKD patients requiring dialysis in China [9]. 30–40% of dialysis-dependent CKD patients died of cardiogenic causes such as acute coronary syndrome or heart failure. However, the preferential option of revascularization for patients with CAD complicated CKD had been controversial. The recommendation of CABG or PCI in the general population was difficult to apply to dialysis patients. Part of the reason was almost all studies with rigorous design and evaluation of coronary revascularization in patients with CAD had ruled out dialysis patients. Another reason was that there were few studies comparing the relative long-time outcomes of DES-PCI with CABG in dialysis patients, and DES were the most commonly used stents nowadays. Several retrospective cohort studies have compared PCI and CABG in dialysis patients [10–12]. The conclusion was that the 3-month mortality was lower in PCI group, however, after that the risk of revascularization and death was higher in PCI group than in CABG group. Overall evidence suggested that dialysis patients generally had a higher risk of long-term cardiac events and/or death after PCI than after CABG [13–15].

The characteristics of coronary disease in CKD patients were labeled as multiple-vessel disease including left main coronary artery, calcification, diffused vessel disease, and small vessel disease. All these characters contributed to huge obstacles in the PCI procedure, especially in the PTCA and BMS era, and often led to failure or insufficient post-expansion after stenting. Nowadays, advanced technology has granted us new tools to deal with these calcified lesions, including cutting balloon, rotablation, and laser ablation. The use of IVUS/OCT gave us visions inside the vessel and improved post-expansion after stenting. All these advances may improve the survival and alter the option of revascularization for this group of patients. As shown in our study, compared with CABG, PCI with DES was non-inferior if not superior in reducing all-cause mortality (PCI 40.7% vs. CABG 50.0%) in our follow-up time, but the difference was not statistically significant (*p* = .37). The two revascularization procedures also showed no difference in outcomes composite of long-term mortality, myocardial infarction, stroke and repeat revascularization, and the survival rate after 19.3 months in this study was 50–60% compared to 40–45% in former studies [4,16,17]. The reason was unclear. That may be partly due to the use of new technology listed above including IVUS, OCT, rotablation, and so on. Another reason may be the highly improved health-care system including multiple disciplinary team (MDT) and follow-up clinic carried out by both cardiologist and nephrologist in our department. The higher survival may be even undermined by the high rate of acute coronary syndrome patients (90.2%) in our study, due to which almost all procedures were performed in acute indication, which can be associated with higher mortality and morbidity. The use of secondary prevention drugs seems to be different between two groups, namely the higher usage of RAAS inhibitor and statin in PCI patients in our study. Although the benefit of statin was quite clear in general population and even in CKD patients, it remains to be controversial in dialysis patients. Both the 4D and Aurora trial failed to show benefit in cardiovascular events from statin in dialysis group, but a large cohort showed statin could reduce mortality in dialysis patients after acute myocardial infarction [18]. Conflicting results also existed regarding to the benefit of RAAS inhibitor on clinical outcomes in dialysis patients. However, according to recent researches, treatment with RAAS inhibitor seems to improve survival and reduce cardiovascular events in dialysis patients [19,20], thus the benefit of PCI group may be partially explained by the higher usage of statin and RAAS inhibitor. In fact, 54.2% patients in average died from non-cardiac morbidities in both groups, which indicated that systemic care may contribute to the improved long-term survival. When it came to the time of these non-cardiac deaths, one out of 17 in PCI group and two out of nine in CABG group were within 30 days during hospitalization after the procedure. Considering the complex comorbidity, most deaths (69.2%) in CABG group were caused by other internal medical diseases like gastro-intestinal bleeding, stroke, infection, and respiratory failure.

The older (≥65 years old) dialysis patients faced 2–3 folder higher mortality in our study. First, the cardiac and pulmonary function declined with increasing age, and these patients also had a higher prevalence of brain vascular disease. All of above increased the risk of anesthesia and perioperative accidents. Second, the older patients were always complicated with more chronic internal medicine diseases, as mentioned before, which contributed to many deaths in this study. Third, giving the poor vessel condition both in coronary and peripheral, the efforts to fully revascularize or apply artery graft frequently failed. At last, the poor prognosis may result from poor compliance due to increased adverse reactions in the elder patients.

Our study described the significant difference in survival rate after revascularization between different dialysis modality for the very first time. First, due to potential risk of volume overload during HD, PD may be preferred in patients with cardiac dysfunction, which means PD patients may have worse heart function from the beginning. In our study, we included LVEF in the multivariate Cox regression to distinguish left ventricle dysfunction, but LVEF-preserved heart failure was also very common in PD patients. We may underestimate the presence of heart failure with normal ejection fraction by using only LVEF. Second, in congestive heart failure (CHF) patients, HD got higher efficiency in removing the excess fluid. In contrast, fluid removal was less predictable and could be inadequate in PD patients. According to a prospective cohort study [21] performed in ESRD patients, mortality risk was higher with PD than with HD among patients with CHF. Lastly, increased risk may be explained by differences in medical monitoring and prescription adaptation related to home care in PD patients.

There were some obvious limitations of our study. First, it is a small sample sized, retrospective, single center study, which makes the proof of the evidence less powerful. Second, there was a significant difference in the prevalence of LM disease between groups and we cannot perform further statistical analysis like propensity score match to eliminate the effects of this diversity due to the small sample size. In fact, the CABG group showed a trend of lower mortality and MACE after 5 years in our follow-up. If we expand the sample size, it is likely to get similar results with previous studies.

In conclusion, among patients with dialysis-dependent CKD, PCI with DES may result in a similar long-term outcome compared with CABG. However, higher mortality was revealed in patients over 65 years and underwent PD. The dilemma needs to be clarified in further large randomized controlled clinical trials.
